# What is the cause of this synchronous palpable abdominal mass in a woman recently diagnosed with lung cancer as demonstrated in Figure 1?

**DOI:** 10.1002/ccr3.1101

**Published:** 2017-07-30

**Authors:** Gerard Feeney, Emer O'Connell, Mike Flood, Cyril Rooney, Fadel Bennani, Kevin Barry

**Affiliations:** ^1^ Department of Surgery Mayo University Hospital Castlebar Ireland; ^2^ Department of Respiratory Medicine Mayo University Hospital Castlebar Ireland; ^3^ Department of Histopathology Mayo University Hospital Castlebar Ireland

**Keywords:** Colonic lesion, general surgery, lung cancer, pathology, radiology, respiratory medicine

## Abstract

Colonic metastases from lung cancer are rare [1, 2]. Presentation of an abdominal mass in the setting of a new lung cancer diagnosis should prompt complete evaluation including endoscopic and CT imaging. This case also highlights the need for immunohistochemical analysis of unusual tumor deposits facilitating appropriate treatment.

CT imaging confirmed a colonic lesion during staging investigations of a 56‐year‐old female smoker with lung adenocarcinoma and mediastinal lymphadenopathy (Fig. 1). Immunohistochemistry confirmed this submucosal lesion was consistent with a metastatic tumor deposit from the known lung primary as biopsies were TTF‐1 and CK7 positive (Figs 2 and 3) 1, 2.

**Figure 1:**
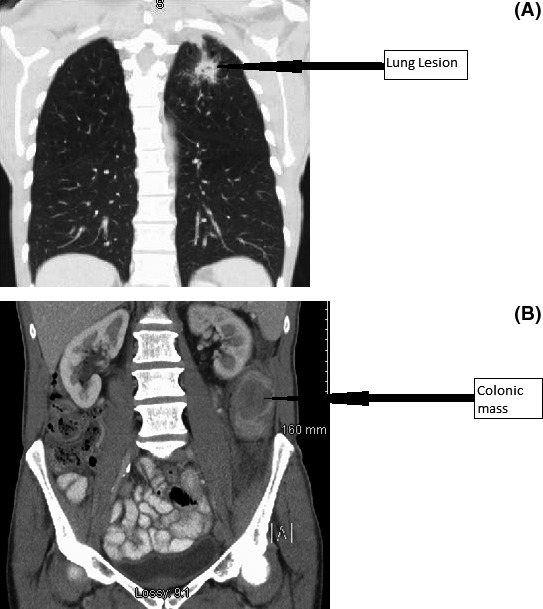
(A) Computed Tomography Thorax – lung lesion. (B) Computed Tomography Abdomen – abdominal lesion.

**Figure 2:**
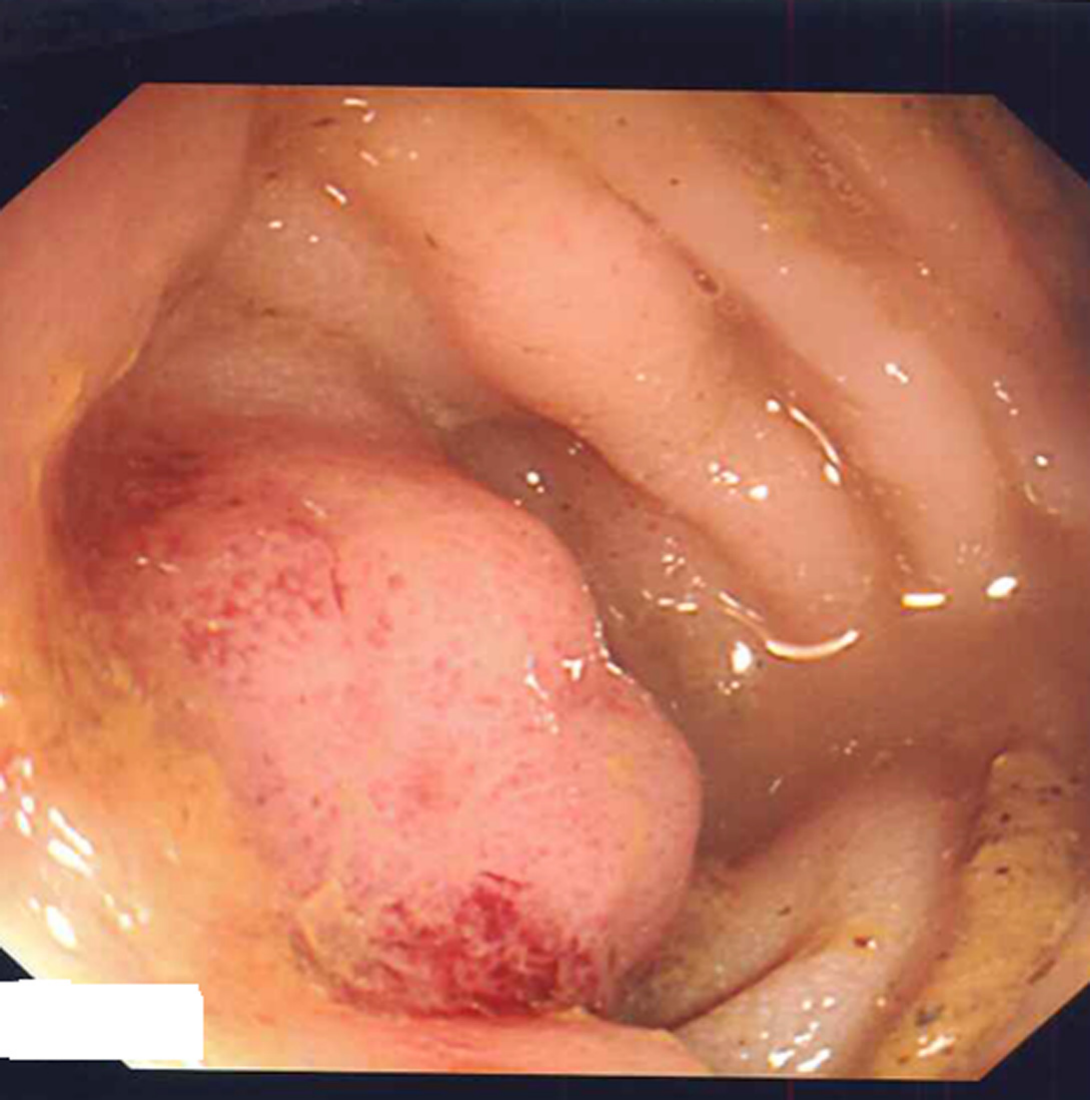
Endoscopic appearance of submucosal metastatic tumour deposit.

**Figure 3:**
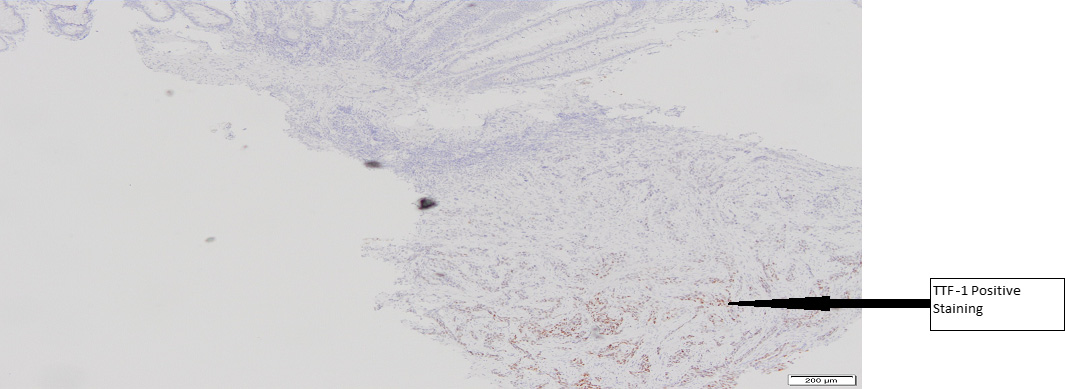
Submucosal tumour deposit staining positive for TTF‐1.

## Authorship

GF: drafted submission and submitted case report for publication. EO: performed literature review and assisted with drafting of submission. MF: obtained and prepared radiology and endoscopic images for case report. CR: is respiratory physician responsible for diagnosing and managing patient's lung adenocarcinoma. FB: is pathologist who reported on colonic biopsy samples and who obtained pathology slides contained in this case report. KB: is general surgeon responsible for investigating patient's colonic lesion through performing colonoscopy and obtaining colonic biopsy samples, assisted with drafting of submission and literature review.

## Conflict of Interest

None declared.
